# Subcutaneous calcinosis: a rare complication of chronic hypercalcemia

**DOI:** 10.11604/pamj.2019.32.76.18187

**Published:** 2019-02-12

**Authors:** Fatima Zahra Zaher, Nawal El Ansari

**Affiliations:** 1 Department of Endocrinology, Diabetology and Metabolic Diseases, University Hospital of Marrakech, Marrakech, Morocco

**Keywords:** Subcutaneous calcinosis, hypercalcemia, primary hyperparathyroidism

## Image in medicine

Subcutaneous calcinosis is a rare entity that may be secondary to either tissue damage, phosphocalcic disturbance or idiopathic origin. In the context of hypercalcemia, subcutaneous calcinosis remains a rare complication with few cases reported in the litterature. We report the case of a 27-year-old patient admitted for chronic hypercalcemia with right femur fracture and renal failure. Clinical examination found hard, painless, non-inflammatory subcutaneous nodules at the 2 elbows and at the lower gum (Panel A), the elbow radiography showed radiopaque structures in favor of subcutaneous calcinosis (Panel B). The etiological investigations of hypercalcemia showed a parathyroid hormon at 2200ng/ml (15-65), with cervical ultrasound showing a right inferior parathyroid nodule of 3cm * 1.6cm, in favor of primary hyperparathyroidism. The evolution was marked by the decrease of the size of the lesions in 15 days after normalization of the phosphocalcic balance after a symptomatic treatment based on intravenous rehydration, diuretics, corticosteroid bolus and bisphosphonate. Although hypercalcemia can reach very high serum levels in primary hyperparathyroidism, it is rarely accompanied by subcutaneous calcinosis. Indeed, it is hyperphosphoremia which represents the main factor involved in the development of cutaneous calcinosis in the context of phosphocalcic disturbances, and this independently of the level of hypercalcemia. Lesions tend to resolve spontaneously when calcium and phosphate levels normalize.

**Figure 1:**
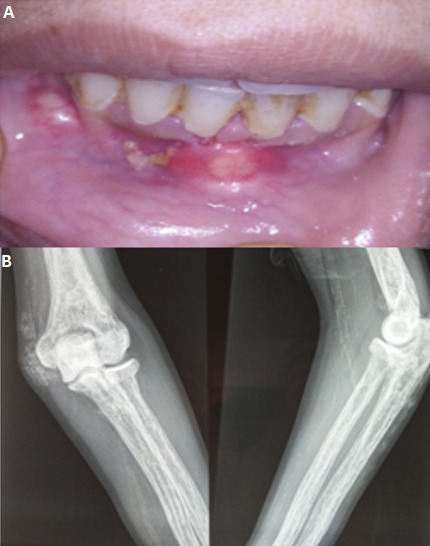
Subcutaneous calcinosis: (A) calcifications of the lower gum; (B) radiological appearance of subcutaneous calcifications of the elbow

